# Die Lübecker Skala der Basis-Mobilität: Assessment schwer in ihrer Mobilität beeinträchtigter geriatrischer Patienten

**DOI:** 10.1007/s00391-023-02220-0

**Published:** 2023-08-14

**Authors:** Sonja Krupp, Robert Wentzel, Friedrich Balck, Martin Willkomm, Jennifer Kasper

**Affiliations:** 1 Forschungsgruppe Geriatrie Lübeck, Krankenhaus Rotes Kreuz Lübeck Geriatriezentrum, Marlistr. 10, 23566 Lübeck, Deutschland; 2 Abteilung Psychosoziale Medizin und Entwicklungsneurowissenschaften, Med. Fakultät Carl Gustav Carus, Technische Universität Dresden, Dresden, Deutschland

**Keywords:** Geriatrisches Assessment, Motorik, Testgüte, Timed-up-and-go-Test, Bodeneffekt, Geriatric assessment, Motor function, Test quality, Timed up & go test, Floor effect

## Abstract

**Hintergrund:**

Im geriatrischen Assessment der Mobilität wird oft der Timed-up-and-go(TUG)-Test eingesetzt. Viele stationäre Patienten können diesen aber nicht bewältigen. Als Performance-Test für diese Zielgruppe wurde die Lübecker Skala der Basis-Mobilität (LSBM) entwickelt.

**Ziel:**

Die Studie untersuchte Eigenschaften der 7 Aufgaben umfassenden LSBM, die auf Item-Ebene eine an die 5‑stufige Bewertung von Beeinträchtigungen gemäß der ICF angelehnte Skalierung aufweist.

**Material und Methoden:**

Bei 77 Patienten, die bei akutgeriatrischer Klinikaufnahme den TUG-Test nicht bewältigt hatten, wurde im Abstand von 7 bis 18 Tagen (t_0_, t_1_) die LSBM absolviert, davon einmal durch 2 Untersucher bewertet. Für die konvergente Validität wurde der De Morton Mobility Index (DEMMI) eingesetzt.

**Ergebnisse:**

Der LSBM-Score und der DEMMI-Score korrelierten hoch (−0,880, *p* < 0,001). Ein Bodeneffekt trat mit der LSBM nicht auf, mit dem DEMMI bei 5 Patienten (6,5 %). Die prädiktive Validität für die Voraussage der Bewältigung des TUG-Tests bei Entlassung aufgrund des Summenscores zu t_0_ betrug für die LSBM −0,577, für den DEMMI 0,542 (Korrelation nach Spearman, *p* = 0,001). Die Interrater-Reliabilität der LSBM lag bei 0,983 (*p* < 0,001), die Korrelation zwischen Test und Retest bei 0,836 (*p* < 0,001), die interne Konsistenz über Cronbachs α bei 0,876. Die LSBM korrelierte mit der physiotherapeutischen Verlaufsbeurteilung (0,482, *p* < 0,001), die Effektstärke als Maß für die Änderungssensitivität lag bei Cohen’s d 0,711.

**Diskussion:**

Die LSBM erleichtert das Setzen von Therapiezielen und ermöglicht die standardisierte Dokumentation bereits kleiner Verbesserungen und Verschlechterungen bei Patienten mit reduzierter Basis-Mobilität.

## Abstract (en)

**Background:**

In the geriatric assessment of mobility, the timed up and go (TUG) test is often used; however, many inpatients are unable to master this test. The Lübeck Scale of Basic Mobility (LSBM) was developed as a performance test for this target group.

**Objective:**

The study investigated the properties of the 7‑task LSBM, which has a scaling at item level based on the 5‑level assessment of impairments according to the ICF.

**Material and methods:**

In 77 patients who had not mastered the TUG test at acute geriatric hospital admission, the LSBM was completed at intervals of 7–18 days (t_0_, t_1_), including one rating by 2 investigators. For convergent validity, the De Morton Mobility Index (DEMMI) was used.

**Results:**

The LSBM score and DEMMI score were highly correlated (−0.880, *p* < 0.001). A floor effect did not occur with LSBM and occurred with DEMMI in 5 patients (6.5%). The predictive validity for predicting coping with TUG test at discharge based on the sum score at t_0_ was −0.577 for the LSBM, and 0.542 for the DEMMI (Spearmanʼs correlation, *p* = 0.001). The interrater reliability of the LSBM was 0.983 (*p* < 0.001), the correlation between test and retest was 0.836 (*p* < 0.001) and the internal consistency via Cronbachʼs α was 0.876. The effect size as a measure of change sensitivity was Cohenʼs d 0.711.

**Conclusion:**

The LSBM facilitates treatment goal setting and allows standardized documentation of even small improvements and deteriorations in patients with reduced basic mobility.

Die standardisierte Erhebung der Mobilität ist ein Kernelement geriatrischen Assessments. In Kliniken und Pflegeheimen ist der Anteil an Personen, die den Timed-up-and-go(TUG)-Test [11] nicht bewältigen, so hoch, dass ein alternativer Performance-Test zur Verfügung stehen sollte. Die Lübecker Skala der Basis-Mobilität (LSBM) fokussiert auf diese Personengruppe. Ziel der Studie war die Überprüfung der Validität, Reliabilität und Änderungssensitivität.

Im geriatrischen Assessment ist die Notwendigkeit, unterschiedliche Schweregrade abzubilden, ein Grund dafür, über mehrere Instrumente pro Dimension zu verfügen. Einige weisen in leistungsschwachen Zielgruppen hohe Bodeneffekte oder eine geringe Differenzierung auf bzw. führen dort gehäuft zum Testabbruch.

Für die Basis-Mobilität behauptet sich der TUG-Test trotz Zweifeln an der Prädiktion des Sturzrisikos [1]. Jedoch bewältigt bei Aufnahme etwa die Hälfte stationärer geriatrischer Patienten die Aufgabenkombination nicht [5]. Hier wird ein anderer Test benötigt, um sowohl kleine Fortschritte als auch Verschlechterungen der Basis-Mobilität standardisiert abzubilden.

Die S1-Leitlinie „Geriatrisches Assessment der Stufe 2, Living Guideline“ [6] führt als Performance-Tests, die keine Gehfähigkeit voraussetzen, die Esslinger Transfer Skala (ETS) [12] und den De Morton Mobility Index (DEMMI) [2, 10] auf.

Die ETS differenziert nicht im Leistungsbereich oberhalb personellen Hilfebedarfs. Eine klare Unterscheidung der Stufen H2 und H3 ist stationär kaum möglich, eine ausreichende wissenschaftliche Evaluation nicht zu erwarten.

Der De Morton Mobility Index (DEMMI) weist in manchen Studien kaum einen Bodeneffekt auf, jedoch hängt dieser von der Studienpopulation ab. Die Validierungsstudie erfolgte an 106 Krankenhauspatienten ab 65 Jahren (47,3 % Frauen, 1,9 % aus Pflegeheimen, 44,6 % vor Aufnahme ohne Gehhilfsmittel) mit mittlerem Barthel-Index von 82,5. Trøstrup et al. [14] fanden nur bei 2 von 155 stationären Patienten ab 65 Jahren den Wert 0, Hulsbæk et al. [4] dagegen bei 31 % der 222 geriatrischen Patienten 9 Tage nach operativer Versorgung einer Hüftfraktur, woraus die Autoren den Schluss ziehen, der DEMMI eigne sich eher für Patienten mit besser erhaltener Funktionsfähigkeit („higher functional levels“). In der Validierungsstudie der deutschen Übersetzung durch Braun et al. [2] bewältigten am 2. Tag akutstationärer Versorgung 9 von 62 geriatrischen Patienten (14,5 %) keine der Aufgaben. Angaben zu Ausschlusskriterien, der Geschlechtsverteilung und dem Barthel-Index fehlen hier. 11 der 15 Items werden 2‑stufig bewertet, bei diesen gibt es keine Differenzierung zwischen Patienten, die die Aufgabe unter Hilfsmitteleinsatz bewältigen, ungeschulte oder geschulte Hilfe benötigen oder auch dann nicht schaffen. De Morton et al. [9] berechneten die Änderungssensitivität durch Vergleich mit einer 5‑stufigen klinischen Einschätzung an 57 stationären Patienten. Signifikante Unterschiede zwischen dem DEMMI-Score bei Aufnahme und bei Entlassung fanden sich nur in der Subgruppe (*n* = 35), die sich laut Therapeutenurteil klinisch stark verbessert hatte („much better“). Søndergaard et al. [13] fanden eine moderate Änderungssensitivität des DEMMI, allerdings untersuchten sie 250 Rehabilitanden nach ihrer Krankenhausentlassung, keine akutgeriatrischen stationären Patienten.

## Methodik

Für die monozentrische longitudinale Studie wurden nach positivem Votum der Ethikkommission der Universität zu Lübeck (Aktenzeichen 20-356) stationäre Patienten des Krankenhaus Rotes Kreuz Lübeck Geriatriezentrum rekrutiert. Der die Datenerhebung durchführende Physiotherapeut fragte seine behandelnden Kollegen stationsweise an etwa 2 Tagen wöchentlich, welche der innerhalb der letzten 4 Tage aufgenommenen Patienten den TUG-Test nicht bewältigt hatten und die weiteren Einschlusskriterien erfüllen (nichtinfektiöse Patienten ohne Ruhedyspnoe mit ausreichender Fähigkeit zur verbalen Kommunikation). Danach informierte er die prospektiv Teilnehmenden und bat sie um ihre schriftliche Einwilligung.

### Lübecker Skala der Basis-Mobilität

Es sollte ein Test für schwer in ihrer Mobilität beeinträchtigte Patienten entwickelt werden, dessen Ergebnisse für Therapie und Pflege unmittelbar relevant sind. Dazu gehört die Überprüfung, inwieweit eine Person in der Lage ist, sich im Bett zu drehen, es zu verlassen und im Zimmer zu gehen. Der Test sollte eine an die Internationale Klassifikation der Funktionsfähigkeit, Behinderung und Gesundheit (ICF) [15] angelehnte Skalierung verwenden, nicht mehr als 10 min und kaum Material erfordern sowie einfach erlernbar sein.

Die in Tab. 1 genannte Skalierung wurde in Abstimmung mit Physiotherapeuten und Pflegefachkräften festgelegt. Zur Visualisierung von Schweregrad (S) 2 wird empfohlen sich vorzustellen, eine gesunde 70-jährige Frau werde vom Patienten spontan um Hilfe gebeten – die Unterstützung sollte ohne Vorinformationen, besondere Körperkräfte oder Techniken möglich sein.

**Table 1:** 

Kodierung des Grades der Einschränkung der Funktionsfähigkeit gemäß ICF	Kodierung des Schweregrades der Einschränkung der Basis-Mobilität bezogen auf die LSBM
ICF xxx.0 0–4 % Problem nicht vorhanden	LSBM S0 Keine Beeinträchtigung
ICF xxx.1 5–24 % Problem leicht ausgeprägt	LSBM S1 Selbstständig (keine Aufsicht erforderlich), aber beeinträchtigt (nur mit Hilfsmitteln, unter Schmerzen, unter großer Anstrengung oder extrem langsam)
ICF xxx.2 25–49 % Problem mäßig ausgeprägt	LSBM S2 Hilfe/Aufsicht durch eine nichtgeschulte Person erforderlich und ausreichend
ICF xxx.3 50–95 % Problem erheblich ausgeprägt	LSBM S3 Hilfe/Aufsicht durch eine geschulte Person erforderlich und ausreichend
ICF xxx.4 96–100 % Problem voll ausgeprägt	LSBM S4 Hilfe gemäß S3 reicht nicht, um Aufgabe auszuführen

*ICF* Internationale Klassifikation der Funktionsfähigkeit, Behinderung und Gesundheit, *LSBM* Lübecker Skala der Basis-Mobilität, *S* Schweregrad

Für das Erkennen von Therapiebedarf und Monitoring des Verlaufs ist die Item-Ebene entscheidend. Dem Wunsch nach Übermittlung eines Gesamtwertes für die Basis-Mobilität wird dadurch Rechnung getragen, dass für jedes der 7 Items dem vorliegenden Schweregrad der entsprechende Punktwert zugeordnet wird (S0–S4 entspricht 0 bis 4 Punkten) und die Punktwerte addiert werden (LSBM-Score 0–28, Angabe z. B. 19/28 = Einschränkung der Basis-Mobilität 68%).

Tab. 2 zeigt die Aufgabenstellung und Bewertung anhand eines konkreten Fallbeispiels. Die Liegefläche sollte eben sein; ein Kopfkissen ist erlaubt. Gelingt das selbstständige Drehen dem Patienten nur unter Festhalten z. B. am Bettgitter, so stellt dies eine Kompensation dar (S1). Obwohl der physiologische Zeitbedarf für die 5 Transfers unterschiedlich ist, wurde das Limit für alle Aufgaben auf 60 s festgelegt, um den Lernaufwand zu minimieren. Das Halten der freien Sitzposition entsprechend S0 (Aufgabe C) kann am einfachsten bewertet werden, wenn der Patient die Arme verschränkt.

**Table 2:** 

Aufgabe		S
A	Transfer Rückenlage → Seitenlage (60–120° zwischen Becken und Unterlage) in max. 60 s	1
B	Transfer Liegen (z. B. Seitenlage) → Sitz auf der Bettkante in max. 60 s	3
C	Sitz ohne Anlehnen (z. B. auf der Bettkante) bei aufgesetzten Füßen mind. 60 s, b. B. (≙ S1 oder höher) mit abgestützten Händen	2
D	Transfer Sitz auf der Bettkante → Sitz auf dem (Roll‑)Stuhl mit Armlehnen in max. 60 s (Sitzhöhe des Bettes ≙ Sitzhöhe des Stuhls, günstigste Position des Stuhls)	3
E	Transfer Sitz auf dem (Roll‑)Stuhl mit Armlehnen → Stand in max. 60 s	3
F	Stehen mind. 60 s, b. B. (≙ S1 oder höher) mit Abstützen der Hände z. B. am Bettrahmen, Handlauf, Gehhilfen	3
G	Stand → 3 m Gehen und Rückkehr zum Ausgangspunkt in max. 60 s, b. B. (≙ S1 oder höher) an Gehhilfen (z. B. Rollator)	4
LSBM-Score		19

*S* Schweregrad

Die Inhaltsvalidität ist bereits durch die Verwendung von Komponenten des TUG-Tests und DEMMI gegeben, sowie dadurch, dass die Items „einen Ausschnitt aus dem Verhaltensbereich darstellen, über den eine Aussage getroffen werden soll“ [8]. Es kann eine beliebige Reihenfolge gewählt werden, bei im Bett befindlichen Patienten bietet sich aber die alphabetische Abfolge an. Ein gesunder Mensch könnte die gesamte Performance in 3 min durchlaufen, in der Zielgruppe ist mit etwa 7 min sowie 3 für die Vorbereitung und Dokumentation zu rechnen. Die Praktikabilität bestätigte sich im Einsatz der LSBM in den Präventionsprojekten POLKA [7] und PfleBeO in Pflegeeinrichtungen, inzwischen auch in der Akutgeriatrie. In den Literaturangaben ist ein Hinweis auf online verfügbare Schulungsvideos enthalten [3].

### Datenerhebung und Statistik

Daten zu Alter, Geschlecht, Barthel-Index, die Mobilität beeinflussenden Erkrankungen und dem kognitiven Status wurden der elektronischen Patientenakte entnommen. Die Kognition wurde für Berechnungen, basierend auf den im Arztbrief aufgeführten Diagnosen und Befunden (Testergebnisse, klinische Beobachtungen), von einem für die LSBM verblindeten Arzt festgelegt auf „kein Hinweis auf Störung der Kognition“: 0, „(Verdacht auf) mindestens leichte kognitive Störung“: 1 und „deutliche Störung der Kognition“: 2. Für die konvergente Validität der LSBM wurde der DEMMI durchgeführt. In mindestens einwöchigem Abstand wurde eine 2. Erhebung der LSBM vorgenommen, am gleichen Tag der aktuell behandelnde verblindete Physiotherapeut gebeten, eine Bewertung der Veränderung der Mobilität unter Therapie vorzunehmen, wobei dem Urteil für Berechnungen die Werte „viel schlechter“: −2, „etwas schlechter“: −1, „unverändert“: 0, „etwas besser“: 1 und „viel besser“: 2 zugeordnet wurden.

Die Datenanalyse erfolgte mit SPSS Version 29 (IBM SPSS Statistics for Windows, Armonk, NY, USA). Die Stichproben wurden mit Mittelwert, Standardabweichung und Streuung beschrieben. Das Signifikanzniveau wurde auf α = 0,05 festgelegt. Mittelwerte wurden bei nicht normal verteilten rangskalierten Daten mittels Mann-Whitney-U-Test verglichen, bei normal verteilten Daten mittels *t*-Test. Korrelationen (z. B. für den Einfluss von Geschlecht, Alter und Kognition auf das Ergebnis der LSBM) wurden nach Spearman berechnet. Für die prädiktive Validität wurden Mittelwertvergleiche mittels *t*-Test vorgenommen und Korrelationen zwischen LSBM bzw. DEMMI zu t_0_ und dem Bewältigen des TUG-Tests bei Klinikentlassung berechnet. Für die Änderungssensitivität wurde neben der Effektstärke gemäß Cohens d die Änderung des LSBM-Scores mit dem Urteil der Physiotherapeuten über den klinischen Verlauf verglichen. Eine Faktorenanalyse (Varimax) erfolgte unter Überprüfung des Kaiser-Meyer-Olkin-Kriteriums, der Sphärizität mittels Bartlett-Test sowie des Kaiser-Guttman-Kriteriums zur Analyse der Dimensionalität und Verteilung der Faktorenladungen auf die Items der LSBM.

## Ergebnisse

Die Untersuchungen erfolgten zwischen dem 03.09.2020 und dem 03.08.2021. Tragischerweise sind sämtliche Daten der Personen, die noch vor der 2. Untersuchung entlassen worden waren oder aus anderen Gründen nicht daran teilnahmen, verloren gegangen. Tab. 3 beschreibt die Gruppe aller an den korrespondierenden Tagen aufgenommenen Patienten, der Subgruppe von Personen, die den TUG-Test nicht bewältigten (48,0 %), und der Probanden, die für beide Untersuchungszeitpunkte zur Verfügung standen. Der Mittelwertvergleich zwischen den letztgenannten Gruppen zeigt keinen signifikanten Unterschied für die Geschlechtsverteilung und das Alter, jedoch für den Barthel-Index (*p* = 0,001). Dieser lag bei den Probanden 5,1 Punkte über dem der ganzen Gruppe der Patienten, die den TUG-Test nicht bewältigten, aber 4,6 Punkte unter dem aller aufgenommenen Patienten.

**Table 3:** 

	Alle Patienten	TUG-Test nicht bewältigt	Probanden
	*n* = 623	*n* = 299	*n* = 77
Frauen/Männer	406/217 (65,2 %/34,8 %)	187/112 (62,5 %/37,5 %)	46/31 (59,7 %/40,3 %)
Alter in Jahren M ± SD (Range)	82,2 ± 7,1 (51–100)	82,6 ± 7,1 (51–100)	82,0 ± 7,6 (51–95)
Barthel-Index M ± SD (Range)	49,5 ± 16,4 (0–100)	39,8 ± 15,7 (0–85)	44,9 ± 14,7 (15–85)

*M* Mittelwert, *SD* Standardabweichung

Als die Mobilität limitierende Erkrankungen lagen u. a. 28-mal Frakturen vor, 28-mal zentralnervös bedingte Bewegungsstörungen, 9‑mal Implantationen einer Endoprothese im Bereich unterer Extremitäten (ohne Fraktur), 5‑mal Herzinsuffizienz und 5‑mal Sarkopenie. 29 Probanden (37,7 %) waren kognitiv unauffällig, bei 20 (26,0 %) bestand der Verdacht auf eine mindestens leichte kognitive Störung, und bei 28 (36,4 %) fanden sich deutliche kognitive Defizite.

### Ergebnisse der LSBM

Ein Patient (1,3 %) wies bereits bei der ersten Untersuchung (t_0_) in allen 7 Items den Schweregrad 0 auf, zum Kontrollzeitpunkt (t_1_) nach im Mittel 9 (7 bis 18) Tagen 2 Patienten (2,6 %). Ein Bodeneffekt wurde bei der LSBM nicht beobachtet, beim DEMMI bei 5 Patienten (6,5 %).

Geschlecht (*p* = 0,324), Alter (*p* = 0,214) und Kognition (*p* = 0,415) korrelierten nicht signifikant mit dem Ergebnis der LSBM.

Tab. 4 weist die Häufigkeitsverteilung der Schweregrade auf die Items, bezogen auf beide Messzeitpunkte, aus.

**Table 4:** 

Schweregrad	S0		S1		S2		S3		S4	
Item	Untersuchungszeitpunkt									
	t_0_	t_1_	t_0_	t_1_	t_0_	t_1_	t_0_	t_1_	t_0_	t_1_
A (Rückenlage – Seitenlage)	27	37	39	37	4	2	5	0	2	1
B (Liegen – Sitzen)	25	32	32	30	6	9	13	6	1	0
C (Sitzen Bettkante)	47	53	17	17	3	2	10	5	0	0
D (Umsetzen Bett – Stuhl)	14	23	21	23	5	10	30	19	7	2
E (Aufstehen)	13	24	27	24	2	3	27	21	8	5
F (Stehen)	8	13	26	28	1	3	22	24	20	9
G (3 m Gehen und zurück)	3	4	12	28	6	7	29	18	27	20

Die Interrater-Reliabilität, überprüft durch simultane Bewertung der Durchführung bei 62 Patienten durch 2 für die Ergebnisse des jeweils anderen verblindete Untersucher, erreichte einen hohen Wert (r = 0,983, *p* < 0,001). Die Korrelation der LSBM-Scores zu t_0_ und t_1_, berechnet auf der Basis der Untersuchungen an 77 Patienten, betrug 0,836 (*p* < 0,001) – wegen des im Mittel 9‑tägigen Intervalls ist davon auszugehen, dass die wahre Retest-Reliabilität oberhalb dieses Wertes liegt. Die interne Konsistenz lag mit Cronbachs α 0,876 ebenfalls hoch.

In der Hauptkomponenten-Analyse fanden sich 2 Faktoren mit Eigenwert über 1, die kumulativ 73,7 % der Varianz erklärten (Summen der quadrierten Ladungen), der Hauptfaktor 57,9 %, der zweite weitere 15,7 %. Der erste Faktor erreichte in der Komponentenmatrix den höchsten Wert beim Transfer vom Sitz in den Stand und könnte Extension repräsentieren, der zweite überwiegt ausschließlich bei der Drehung von der Rücken- in die Seitenlage und könnte der Rumpfrotation entsprechen.

Für die Korrelation des LSBM-Scores mit der Bewertung der Items „Aufsetzen und Umsetzen“ (−0,547, *p* < 0,001) und „Aufstehen und Gehen“ (−0,537, *p* < 0,001) des Barthel-Index sowie des Summenscores (−0,535, *p* < 0,001) fanden sich moderate bis hohe Werte. Mit −0,880 (*p* < 0,001) bestand eine hohe Korrelation des LSBM-Scores mit dem DEMMI-Score und Rohwert. Tab. 5 weist für jeden der 77 Patienten den Summenscore der LSBM im Vergleich zu dem des DEMMI aus, wobei die Anzahl des Auftretens der Kombinationen angegeben ist. Zum Beispiel fand sich bei drei Patienten mit Summenscore 0 im DEMMI ein Summenscore von 25 in der LSBM (Einschränkung der Basis-Mobilität 25/28 = 89 %).

**Table 5:** 

28	–	–	–	–	–	–	–	–	–	–	–	–	–	–	–	–	–	–	–	–
27	–	–	–	–	–	–	–	–	–	–	–	–	–	–	–	–	–	–	–	–
26	–	–	–	–	–	–	–	–	–	–	–	–	–	–	–	–	–	–	–	–
25	3	–	–	–	–	–	–	–	–	–	–	–	–	–	–	–	–	–	–	–
24	–	–	–	–	–	–	–	–	–	–	–	–	–	–	–	–	–	–	–	–
23	1	–	–	–	–	–	–	–	–	–	–	–	–	–	–	–	–	–	–	–
22	–	–	–	–	–	–	–	–	–	–	–	–	–	–	–	–	–	–	–	–
21	–	4	–	–	–	–	–	–	–	–	–	–	–	–	–	–	–	–	–	–
20	–	1	–	–	–	–	–	–	–	–	–	–	–	–	–	–	–	–	–	–
19	1	2	–	–	–	–	–	–	–	–	–	–	–	–	–	–	–	–	–	–
18	–	2	–	–	–	–	–	–	–	–	–	–	–	–	–	–	–	–	–	–
17	–	1	1	–	1	1	–	–	–	–	–	–	–	–	–	–	–	–	–	–
16	–	1	–	–	–	–	–	–	–	–	–	–	–	–	–	–	–	–	–	–
15	–	–	1	–	3	–	–	–	–	–	–	–	–	–	–	–	–	–	–	–
14	–	1	2	1	–	1	–	–	–	–	–	–	–	–	–	–	–	–	–	–
13	–	1	–	1	3	–	–	–	–	–	–	–	–	–	–	–	–	–	–	–
12	–	–	–	–	1	–	–	1	–	–	–	–	–	–	–	–	–	–	–	–
11	–	–	–	2	–	–	1	–	–	–	–	–	–	–	–	–	–	–	–	–
10	–	–	–	–	–	1	2	–	1	–	–	–	–	–	–	–	–	–	–	–
9	–	–	–	–	–	–	–	–	2	–	–	–	–	–	–	–	–	–	–	–
8	–	–	–	–	–	1	–	1	–	–	1	–	–	–	–	–	–	–	–	–
7	–	–	–	–	–	1	1	–	–	–	1	–	–	–	–	–	–	–	–	–
6	–	–	–	–	1	–	2	–	1	1	–	1	–	–	–	–	–	–	–	–
5	–	–	–	–	–	1	–	–	1	–	–	–	–	–	–	–	–	–	–	–
4	–	–	–	–	–	–	2	–	1	1	–	1	2	1	–	–	–	–	–	–
3	–	–	–	–	–	–	–	–	–	2	–	1	–	–	–	–	–	–	–	–
2	–	–	–	–	–	–	–	–	–	–	–	1	–	–	–	–	–	–	–	–
1	–	–	–	–	–	–	–	–	–	–	–	–	1	1	–	–	–	–	–	–
0	–	–	–	–	–	–	–	–	–	–	–	–	–	1	–	–	–	–	–	–
–	0	1	2	3	4	5	6	7	8	9	10	11	12	13	14	15	16	17	18	19

### Verlauf der Werte und Änderungssensitivität

Den Verlauf der Werte der LSBM gibt Tab. 6 wieder. Die 25., 50. und 75. Perzentile des LSBM-Scores lagen zu t_0_ bei 6, 12 und 17, zu t_1_ bei 3, 8 und 14 Punkten, die Streubreite zu t_0_ bei 0 bis 25, zu t_1_ bei 0 bis 22 Punkten. Der LSBM-Score stieg individuell um bis zu 12 Punkte (Verschlechterung) bzw. fiel um bis zu 16 Punkte ab (Verbesserung). Die Mittelwertdifferenz zwischen t_0_ und t_1_ war signifikant (*p* = 0,001, *t*-Test bei abhängigen Stichproben). Die Effektstärke als Maß für die Änderungssensitivität lag bei einem Cohen’s d von 0,711 (Konfidenzintervall 0,459–0,959). Für die Änderungssensitivität beurteilten außerdem bezüglich der LSBM verblindete Physiotherapeuten die Veränderung der Mobilität von t_0_ zu t_1_ bei 77 Patienten. Die Korrelation dieses Urteils mit der Änderung des LSBM-Scores war moderat bis hoch (0,482, *p* < 0,001). Tab. 7 zeigt die zum Therapeutenurteil korrespondierenden Änderungen des LSBM-Scores. 43 der 45 Patienten, die sich im LSBM-Score um mindestens 2 Punkte verbessert hatten, wurden auch von den sie behandelnden Physiotherapeuten als verbessert eingestuft. Laut Therapeutenurteil hatten sich 3 Patienten verschlechtert und 60 verbessert, gemäß LSBM 6 Patienten (7,8 %) verschlechtert und 54 (70,1 %) verbessert. 31 (40,3 %) konnten den TUG-Test bei Krankenhaus-Entlassung weiterhin nicht bewältigen.

**Table 6:** 

	t_0_ (*n* = 77)	t_1_ (*n* = 77)	Differenz t_0_ → t_1_
	M ± SD	M ± SD	M ± SD
A (Rückenlage – Seitenlage)	0,91 ± 0,95	0,58 ± 0,68	−0,33 ± 0,85
B (Liegen – Sitzen)	1,13 ± 1,09	0,86 ± 0,91	−0,27 ± 0,75
C (Sitzen, Bettkante)	0,69 ± 1,04	0,47 ± 0,84	−0,22 ± 0,79
D (Umsetzen Bett – Stuhl)	1,94 ± 1,33	1,40 ± 1,23	−0,53 ± 0,91
E (Aufstehen)	1,87 ± 1,34	1,47 ± 1,35	−0,40 ± 0,95
F (Stehen)	2,26 ± 1,43	1,84 ± 1,35	−0,42 ± 0,91
G (3 m Gehen und zurück)	2,84 ± 1,18	2,29 ± 1,34	−0,56 ± 0,97
LSBM-Score	11,64 ± 6,39	8,91 ± 6,28	−2,73 ± 3,84

*M* Mittelwert, *SD* Standardabweichung

**Table 7:** 

Klinisches Urteil, Physiotherapeut	Viel schlechter	Etwas schlechter	Unverändert	Etwas besser	Viel besser
Anzahl (% der Patienten)	1 (1,3 %)	2 (2,6 %)	14 (18,2 %)	26 (33,8 %)	34 (44,2 %)
Änderung LSBM-Score M ± SD	12,00 ± 0,00	1,50 ± 3,54	0,36 ± 1,28	−2,65 ± 1,98	−4,44 ± 4,11

*M* Mittelwert, *SD* Standardabweichung

### Prädiktive Validität

Für die prädiktive Validität der LSBM und des DEMMI wurden Mittelwertvergleiche der Gruppe, die den TUG-Test bei Entlassung bewältigte (*n* = 46), und der Gruppe, die dies weiterhin nicht konnte (*n* = 25), vorgenommen (*t*-Test). In der Gruppe, die den TUG-Test zuletzt schaffte, lag der Summenscore der LSBM zu t_0_ bei 9,02 ± 5,19, der des DEMMI bei 6,41 ± 3,47. Der Summenscore der weiterhin „nicht-TUG-Test-fähigen“ Patienten hatte zu t_0_ gemäß LSBM bei 16,84 ± 5,45 gelegen, gemäß DEMMI bei 2,48 ± 2,37 (*p* < 0,001 für beide Mittelwertunterschiede und beide Tests). Die Korrelation (Spearman) zwischen Bewältigung des TUG-Test bei Entlassung und Summenscore zu t_0_ und betrug für die LSBM −0,577 (*p* = 0,001), für den DEMMI 0,542 (*p* = 0,001).

## Diskussion

Die Studie zeigte eine hohe Korrelation der Ergebnisse von LSBM und DEMMI. Kein Patient erreichte das schlechteste Ergebnis der LSBM (Score 28). Somit konnten Verschlechterungen – anders als beim TUG-Test und DEMMI – stets abgebildet werden. Etwa 40 % der Patienten, die den TUG-Test bei Aufnahme nicht bewältigten, erwarben diese Fähigkeit auch nicht. Dagegen konnte die LSBM bei etwa 70 % eine Verbesserung dokumentieren.

Die Berechnung von Summenscores ist bei rangskalierten Tests angreifbar, jedoch üblich (z. B. DEMMI, Barthel-Index). Mit der ICF vertrauten Nutzern kann das arithmetische Mittel der Schweregrade über alle 7 Items der LSBM einen Gesamteindruck von der Schwere der Einschränkung der Basis-Mobilität des Patienten vermitteln. Für die Therapie entscheidend ist jedoch die Betrachtung der Items.

Praktikabilität und Akzeptanz der Erhebung der Basis-Mobilität mittels LSBM haben sich im Rahmen dieser Studie, in der Aufnahme-Routine des akutgeriatrischen Krankenhauses sowie in den Präventionsprojekten POLKA [7] und PfleBeO in Pflegeeinrichtungen erwiesen, in denen geschultes Personal die Erhebung durchführt. Die Notwendigkeit, sich bei der Bewertung der Mobilität eines Krankenhauspatienten oder Pflegeheimbewohners zwischen den Schweregraden 2 und 3 (ungeschulte/geschulte Hilfe erforderlich) zu entscheiden, richtet das Augenmerk auf die Frage, ob Kontaktpersonen der Anleitung zur Unterstützung der Mobilität bedürfen. Solange ein Patient nur eine Funktionsfähigkeit gemäß S3 erlangt hat, ist er zu instruieren, sich durch Personal bei der Ausübung unterstützen zu lassen, bis ggf. An- und Zugehörige, die diese Hilfe ebenfalls leisten möchten, geschult sind. Der Schweregrad 1 erfasst Defizite im Stadium der Kompensation und zeigt Interventionsbedarf zur Prävention eines Verlusts der Selbstständigkeit auf.

Bei kognitiv stark eingeschränkten Patienten ist der TUG-Test oft nicht valide einsetzbar [6], weil der mehrteilige Ablauf nicht verstanden wird. Die LSBM bietet hier den Vorteil einzeln zu bewertender Teilaufgaben, die oft auch durch bloße Beobachtung bewertet werden können.

Die LSBM wurde primär für den Einsatz bei Personen entwickelt, die die im TUG-Test gestellte Aufgabenkombination nicht bewältigen, in der „TUG-Test-fähigen“ Subpopulation geriatrischer Patienten ist mit einem inakzeptablen Deckeneffekt zu rechnen. Durch Berücksichtigung der Dauer der einzelnen Transfers ließe sich dieser Nachteil bereinigen: Eine Studie zur Timed Luebeck Scale of Basic Mobility (t-LSBM) wird derzeit durchgeführt.

### Limitationen

Der Verlust der Daten jener Patienten, die der Studienteilnahme zugestimmt hatten, dann aber nicht für die komplette Datenaufnahme zur Verfügung standen, stellt die bedeutendste Limitation dieser Studie dar. Auch wurde keine Zufallsstichprobe aus sämtlichen Patienten gezogen, sondern der Untersucher vertraute an den ihm möglichen Rekrutierungstagen für das Vorliegen der Einschlusskriterien auf das Urteil der aufnehmenden Physiotherapeuten. Die beschriebene Stichprobe weist jedoch gemäß den in Tab. 1 demonstrierten Daten hinsichtlich Geschlechts- und Altersdurchschnitt keine signifikanten Unterschiede zur Zielgruppe auf; der Barthel-Index liegt, wie zu erwarten, zwischen dem Mittelwert für alle stationären geriatrischen Patienten (einschließlich derer, die den TUG-Test bewältigten) und dem der Gruppe, die die Aufgabenstellung des TUG-Test nicht absolvieren konnte (einschließlich derer, bei denen Ausschlusskriterien wie Ruhedyspnoe vorlagen).

Im Hinblick auf die Retest-Reliabilität wäre ein kürzeres, für die Änderungssensitivität ein längeres Intervall zwischen den beiden Untersuchungszeitpunkten wünschenswert gewesen, eine 3malige Erhebung konnte jedoch nicht realisiert werden. Es muss davon ausgegangen werden, dass die Korrelation für die Retest-Reliabilität bei kleinerem Mess-Intervall noch höher gewesen wäre, die Effektstärke für die Änderungssensitivität bei größerem Intervall.

Eine ungenauere physiotherapeutische Einschätzung des Verlaufs der Basis-Mobilität muss angenommen werden, wenn zwischenzeitlich ein Behandlerwechsel stattgefunden hatte, sowie in Fällen, in denen außerhalb des Bereichs der Basis-Mobilität ein Funktionsgewinn erzielt wurde (z. B. bei der Gehstrecke).

## Fazit für die Praxis

- Die LSBM ist ein valider Performance-Test mit hoher Interrater- und Retest-Reliabilität, interner Konsistenz und Änderungssensitivität.
- Die LSBM korreliert hoch mit dem DEMMI, beschränkt sich jedoch auf 7 Parameter der Basis-Mobilität und stellt den Schweregrad von Funktionseinschränkungen für diese differenzierter dar.
- Durch Einsatz der LSBM lassen sich bei Patienten, die den TUG-Test nicht bewältigen, schon kleine Verbesserungen und Verschlechterungen erfassen und standardisiert dokumentieren.
- Die LSBM ist sowohl für Klinikpatienten als auch in Pflegeeinrichtungen durch geschultes Personal einsetzbar und in 10 min zu erheben.
